# UV-B Induces Chloroplast Movements in a Phototropin-Dependent Manner

**DOI:** 10.3389/fpls.2019.01279

**Published:** 2019-10-15

**Authors:** Paweł Hermanowicz, Agnieszka Katarzyna Banaś, Olga Sztatelman, Halina Gabryś, Justyna Łabuz

**Affiliations:** ^1^Laboratory of Photobiology, Małopolska Centre of Biotechnology, Jagiellonian University, Krakow, Poland; ^2^Department of Plant Biotechnology, Faculty of Biochemistry, Biophysics and Biotechnology, Jagiellonian University, Krakow, Poland; ^3^Institute of Biochemistry and Biophysics, Polish Academy of Sciences, Warsaw, Poland

**Keywords:** phototropin1, phototropin2, chloroplast movements, UV-B, UVR8, *Arabidopsis*

## Abstract

We examined the impact of UV-B irradiation on chloroplast movements in *Arabidopsis* leaves. Directional chloroplast movements induced by blue light have been described in multiple plant species. In weak light, chloroplasts accumulate at periclinal cell walls to increase light capture. In strong light, chloroplasts exhibit the avoidance response, as they move towards anticlinal walls to protect the photosynthetic apparatus from light-induced damage. In *Arabidopsis*, chloroplast movements are triggered by phototropins, phot1 and phot2, which are known as blue/UV-A photoreceptors. We found that irradiation with UV-B of 3.3 µmol·m^−2^·s^−1^ induced chloroplast accumulation in wild-type plants. UV-B-triggered accumulation was dependent on the presence of phototropins, especially phot1, but not on UVR8 (the canonical UV-B photoreceptor). Irradiation with strong UV-B of 20 µmol·m^−2^·s^−1^ did not induce substantial chloroplast relocations in wild-type leaves. However, in the *jac1* mutant, which is defective in chloroplast accumulation, strong UV-B elicited chloroplast avoidance. This indicated that UV-B can also activate signaling to the avoidance response. To assess the possibility of indirect effects of UV-B on chloroplast movements, we examined the impact of UV-B on the actin cytoskeleton, which serves as the motile system for chloroplast movements. While irradiation with UV-B of 3.3 µmol·m^−2^·s^−1^ did not affect the actin cytoskeleton, strong UV-B disrupted its structure as shown using an *Arabidopsis* line expressing Lifeact-green fluorescent protein (GFP). In wild-type plants, pretreatment with strong UV-B attenuated chloroplast responses triggered by subsequent blue light irradiation, further indicating that this UV-B intensity also indirectly affects chloroplast movements. Taken together, our results suggest that the effect of UV-B on chloroplast movement is twofold: it directly induces phototropin-mediated movements; however, at higher intensities, it attenuates the movements in a nonspecific manner.

## Introduction

Apart from visible light, the solar spectrum contains ultraviolet radiation, by convention divided into the UV-A (315–400 nm), UV-B (280–315 nm), and UV-C range (200–280 nm). As UV-C is absorbed by the ozone layer, only UV-A and UV-B reach the Earth’s surface (Aphalo et al., 2012). UV-B is an important environmental cue for plants (Robson et al., 2019), but strong UV-B causes damage to cellular constituents (Schuch et al., 2017). The main plant photoreceptor for UV-B is UVR8 (Jenkins, 2017). The UVR8 absorption spectrum exhibits a band between 260 and 300 nm, with a maximum at 282 nm and a shoulder at 290 nm (Yang et al., 2015). UVR8 is a dimeric protein. Upon UV-B exposure, it monomerizes (Rizzini et al., 2011) and moves from the cytoplasm to the nucleus (Yin et al., 2016). It is involved in protection against damage caused by excessive UV-B radiation through regulation of gene expression related to flavonoid biosynthesis, antioxidant activities, and DNA repair (Yin and Ulm, 2017). UVR8 participates also in UV-B-induced phototropic bending. However, its activity in this reaction is repressed in wild-type (WT) plants. The main receptors for UV-B-induced phototropism are phototropins (Vandenbussche et al., 2014; Vanhaelewyn et al., 2016).

Phototropins are known as blue/UV-A light photoreceptors, which control responses aimed at improving photosynthetic efficiency in plants. Two phototropins, phot1 and phot2, are found in *Arabidopsis thaliana*. They share highly redundant functions. Both control phototropism, stomata opening, chloroplast movements, leaf expansion, and positioning (Christie et al., 2015). In reactions controlled by both phototropins, phot1 is more light sensitive than phot2 (Sakai et al., 2001; Harada and Shimazaki, 2007). Phototropin expression is regulated by light. In mature *Arabidopsis* leaves, *PHOT1* mRNA levels are reduced after blue and red light treatments, in contrast to *PHOT2* mRNA levels, which are elevated (Łabuz et al., 2012). Blue light decreases the level of phototropin1 protein (Sakamoto and Briggs, 2002); however, the expression of phototropin2 at the protein level does not change under similar conditions (Kong et al., 2006). Phot2 is continuously degraded and re-synthesized in darkness. Blue light also causes its degradation, but at a slower rate (Aggarwal et al., 2014). The phototropin molecule consists of two parts, an N-terminal photosensory part with two light, oxygen, and voltage (LOV)-regulated domains and a C-terminal serine/threonine kinase domain. Each LOV domain contains a flavin mononucleotide (FMN) chromophore. Absorption spectra of LOV domains feature a peak in UV-A, around 370–380 nm. In the blue part of the spectrum, the absorption maximum of LOV domains is around 445–449 nm (Salomon et al., 2000; Christie et al., 2002; Kasahara et al., 2002b). Pure FMN in solution is characterized by absorbance maxima in the UV range at 220, 266, 370 nm and in the visible range at 450 nm (Copeland and Spiro, 1986). Light absorption leads to conformational changes in the LOV2 domain that result in the activation of the phototropin kinase domain (Tokutomi et al., 2008). Phototropins are considered as blue/UV-A photoreceptors; however, only recently has it been shown that they mediate UV-B-induced phototropism in etiolated *Arabidopsis* seedlings, in a manner dependent on their kinase activity and NPH3 (NONPHOTOTROPIC HYPOCOTYL3) dephosphorylation (Vanhaelewyn et al., 2016).

Blue light induces directional movements of chloroplasts in plants. In a few species of green algae, mosses, and ferns [e.g., *Mougeotia scalaris* (Suetsugu et al., 2005b), *Physcomitrella patens* (Kadota et al., 2000), and *Adiantum capillus-veneris* (Kawai et al., 2003)], also red light is known to trigger chloroplast relocations. Blue light signaling to chloroplast movements depends on phototropins (Banaś et al., 2012). In weak light, chloroplasts gather at the cell walls perpendicular to the direction of incident light, leading to a decrease in total leaf transmittance. This reaction, called the accumulation response, improves photosynthetic efficiency (Zurzycki, 1955; Gotoh et al., 2018). Chloroplast accumulation is controlled redundantly by both phototropins in *Arabidopsis*. In WT *Arabidopsis*, chloroplasts accumulate at blue light intensities between ∼0.08 and 20 μmol·m^−2^·s^−1^. In the *phot2* mutant, in which only phot1 is active, chloroplasts accumulate at any intensity greater than ∼0.08 μmol·m^−2^·s^−1^ of blue light. In the *phot1* mutant, in which only phot2 is active, chloroplast accumulation occurs in the range of 2–20 μmol·m^−2^·s^−1^ of blue light (Jarillo et al., 2001; Kagawa et al., 2001; Sakai et al., 2001). In strong blue light, chloroplasts position at cell walls parallel to the direction of incident light. This avoidance response results in an increase of leaf transmittance. It protects chloroplasts against photodamage in stress conditions (Kasahara et al., 2002a; Sztatelman et al., 2010). In WT plants, sustained chloroplast avoidance, controlled by phototropin2, occurs at light intensities higher than 20 μmol·m^−2^·s^−1^ of blue light (Sakai et al., 2001). In the *phot2* mutant, only small, transient chloroplast avoidance is observed (Luesse et al., 2010), (Sztatelman et al., 2016). In land plants, chloroplast movements rely predominantly on actin filaments (Kandasamy and Meagher, 1999; Krzeszowiec et al., 2007), in particular cp-actin (chloroplast-actin) (Kadota et al., 2009; Yamashita et al., 2011; Tsuboi and Wada, 2012).

Chloroplast movements induced by the UV radiation were investigated only in a few species of non-vascular plants and aquatic monocots. UV-B induces chloroplast clumping in the aquatic monocot *Halophila stipulacea* (Sharon et al., 2011) and abolishes chloroplast rhythmical movements in the green alga *Ulva petrosa* (Han et al., 1998). The impact of UV on directional chloroplast movements was investigated in the aquatic monocot *Lemna trisulca* and the moss *Funaria hygrometrica*. In *Lemna*, UV irradiation containing the UV-C range induced chloroplast accumulation at low intensities and inhibited the movements at higher intensities (Zurzycki, 1962). In *Funaria*, the action spectrum of chloroplast movements from the dark position to the walls perpendicular to the direction of radiation featured three maxima, around 266, 366, and 454 nm. The peak at 266 nm was the greatest (Zurzycki, 1967).

In this work, we show that in the model plant *A. thaliana*, UV-B induces chloroplast movements in a phototropin-dependent manner. UVR8 is not involved in this response. Irradiation with strong UV-B disrupts the actin cytoskeleton architecture in the epidermal and palisade cells of *Arabidopsis* leaves. In line with this, pretreatment of WT leaves with strong UV-B reduces the magnitude of chloroplast responses to subsequent illumination with blue light.

## Materials and Methods

### Plants and Growth Conditions

WT Col-0 *Arabidopsis*, *phot1*: SALK_088841 (Lehmann et al., 2011), *uvr8-6:* SALK_033468 (Favory et al., 2009), *jac1-3:* WiscDsLox457-460P9 plants were purchased from Nottingham Arabidopsis Stock Centre. The *phot2: npl1-1* mutant (Jarillo et al., 2001) was gifted by Jose Jarillo (Madrid, Spain). Seeds of *Arabidopsis* expressing Lifeact–green fluorescent protein (GFP) (Smertenko et al., 2010) were a gift from Tim Hawkins (Durham, United Kingdom). The *phot1phot2* double mutant was selected from crosses (SALK_088841 with *npl1-1*) (see **Supplementary Figure 1** for genotyping). The *jac1-3* mutant (WiscDsLox457-460P9) was selected in this study (see **Supplementary Figure 2** for line characteristics). Seeds were sown in Jiffy-7 pots (Jiffy Products International AS) and vernalized at 4°C for 2 days. Plants were transferred to a growth chamber (Sanyo MLR 350H) at 23°C, 80% relative humidity, with a photoperiod of 10-h light and 14-h darkness, at 70 μmol·m^−2^·s^−1^ of light supplied by fluorescent lamps (FL40SS.W/37, Sanyo, Japan).

### UV-B and Light Treatments

**Figure 1:**
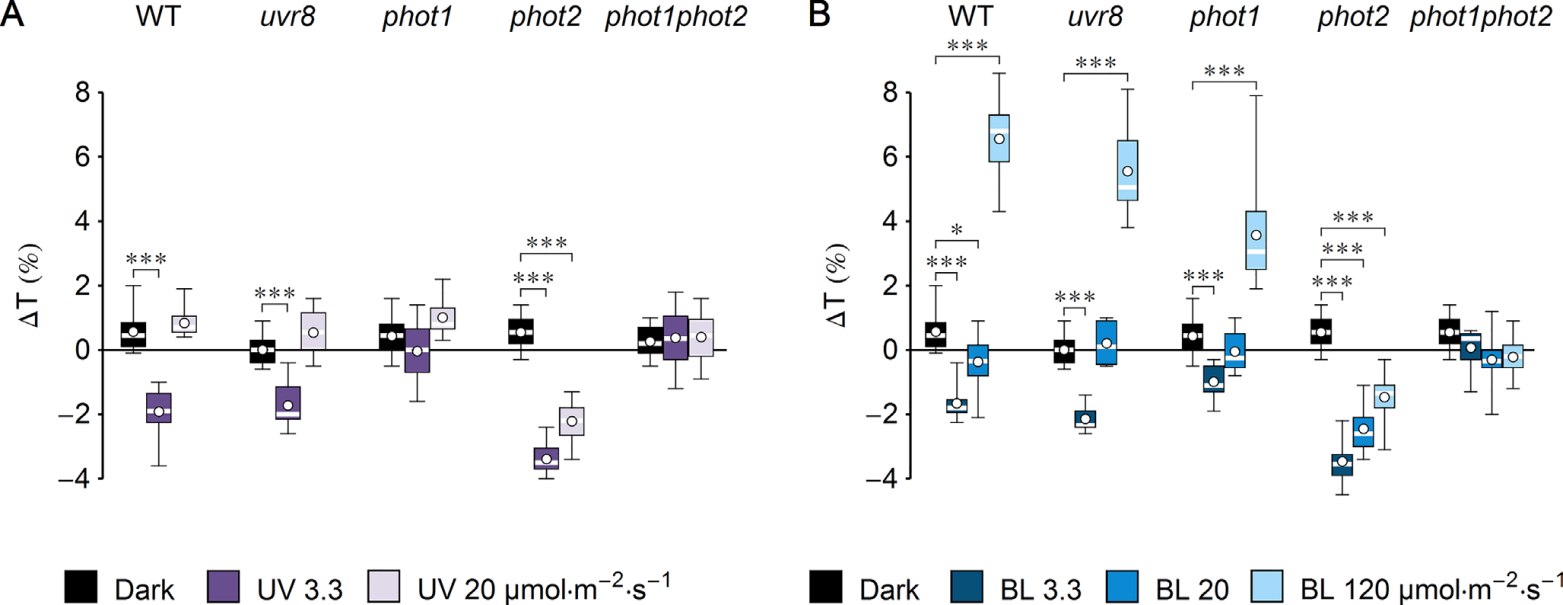
Effect of UV-B and blue light (BL) on leaf transmittance in dark-adapted *Arabidopsis* leaves of wild-type (WT), *uvr8*, *phot1*, *phot2*, and *phot1phot2* mutant plants. **(A)** Leaves were irradiated for 1 h with UV-B (violet boxes) of 3.3 or 20 µmol·m^−2^·s^−1^ or kept in darkness (black boxes). **(B)** Leaves were irradiated for 1 h with blue light (blue boxes) of 3.3 or 20 or 120 µmol·m^−2^·s^−1^ or kept in darkness (black boxes). The difference Δ*T* between leaf transmittance after and before the treatment was measured for red light (660 nm). Plots in **(A)** and **(B)** share the dark controls. The leaves irradiated with UV-B and those irradiated with blue light were taken from the same batch of plants. Asterisks indicate statistically significant differences between means for WT and mutant lines (adjusted *p* values: *0.01 < *p* < 0.05, ***p < 0.001). Each box represents 12 measurements.

**Figure 2:**
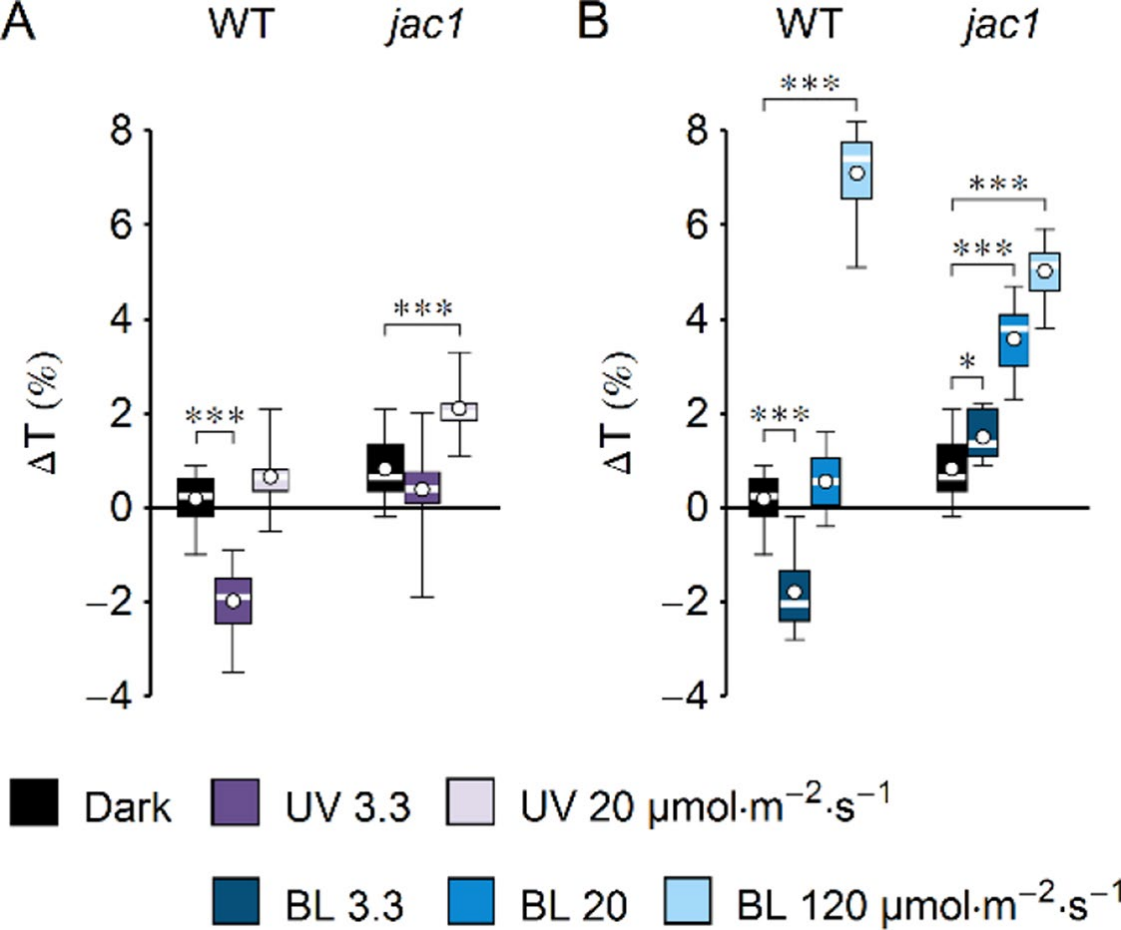
Effect of UV-B and blue light (BL) on leaf transmittance in dark-adapted *Arabidopsis* leaves of wild-type (WT) and *jac1* mutant plants. **(A)** Leaves were irradiated for 1 h with UV-B (violet boxes) of 3.3 or 20 µmol·m^−2^·s^−1^ or kept in darkness (black boxes). **(B)** Leaves were irradiated for 1 h with blue light (blue boxes) of 3.3 or 20 or 120 µmol·m^−2^·s^−1^ or kept in darkness (black boxes). The difference Δ*T* between leaf transmittance after and before the treatment was measured for red light (660 nm). Plots in **(A)** and **(B)** share the dark controls. The leaves irradiated with UV-B and those irradiated with blue light were taken from the same batch of plants. Asterisks indicate statistically significant differences between means for WT and *jac1* (adjusted *p* values: *0.01 < *p* < 0.05, ***p < 0.001). Each box represents 12 measurements.

In experiments on chloroplast movements and the actin cytoskeleton structure, leaves were detached from 4- to 5-week-old plants, dark adapted for at least 16 h. The upper side of leaves was irradiated with UV-B of 3.3 (1.3 W·m^−2^) or 20 µmol·m^−2^·s^−1^ (7.9 W·m^−2^) for 1 h, supplied by USHIO UV-B G8T5E fluorescent tubes. UV-B was filtered through UG-11 (Knight Optical, UK) and ZUS0325 (Asahi Spectra Co, Japan) filters, as well as two layers of cellulose acetate foil (95 µm thick, Rachow Kunststoff-Folien, Germany) (the filtered spectrum shown in **Supplementary Figure 3A**). In the experiments with blue light irradiation (455 nm, LED, LXHL-PR09, Ledium Ltd., Hungary), the intensities of 3.3, 20, or 120 µmol·m^−2^·s^−1^ were used (the spectrum shown in **Supplementary Figure 3B**). Control leaves were given a mock irradiation (kept in the irradiation chamber but covered with aluminum foil). Changes of leaf transmittance at 660 nm, shown in **Figures 1** and **2**, were measured before and immediately after irradiation, with a custom-made photometer (Walczak and Gabrys, 1980). For expression studies, dark-adapted plants were irradiated with UV of 0.2 µmol·m^−2^·s^−1^ for 3 h through WG305 cut-off filters in a growth chamber equipped with USHIO UV-B Lamps G8T5E as in Banaś et al. (2018).

### Photometric Method

Chloroplast movements triggered by blue light were assessed using the photometric method according to Gabryś et al. (2017), which relies on measurements of changes in leaf transmittance. Chloroplast responses to continuous blue light (Luxeon Star Royal Blue LXHL-FR5C LED, 460 nm) of 1.6 or 120 μmol·m^−2^·s^−1^ were measured in detached leaves of overnight dark-adapted 4- to 5-week-old plants. Amplitudes and velocities of chloroplast responses after light treatments were calculated based on the photometric curves, using a custom-written Mathematica (Wolfram Research, US) package.

### Confocal Microscopy

Microscopic observations were performed with the Axio Observer.Z1 inverted microscope (Carl Zeiss, Jena, Germany) and the LSM 880 confocal module. Plan-Neofluar 40×, NA 1.3, objective was used with oil immersion. The actin cytoskeleton was observed in the upper epidermis and the upper parts of palisade cells in 4- to 5-week-old Lifeact-GFP rosette leaves. The Ar laser line of 488 nm was used to excite GFP and chlorophyll. The emission in the range of 494–597 nm was recorded as the green channel. The emission in the range of 647–721 nm was recorded as the magenta channel. Chloroplast positioning was observed in the palisade parenchyma of WT *Arabidopsis* leaves as well as *uvr8*, *phot1*, *phot2*, *phot1phot2*, and *jac1* mutants. The 633-nm He–Ne laser was used to excite chlorophyll, and the emission in the range of 647–721 nm was recorded as the magenta channel.

To examine the effect of UV-B on chloroplast positioning, stacks were collected on the upper surface of WT and mutant leaves, with the interval between slices set to 1.06 μm. Projection images were calculated from slices corresponding to the first 40 μm of stacks, starting from the upper surface of the epidermis. This range included the epidermis and upper parts of palisade cells. Images were then segmented using Otsu’s thresholding in ImageJ. The fraction of the image area occupied by chloroplasts was used as a measure of chloroplast relocation in response to irradiation. To examine the effect of UV-B on the actin cytoskeleton, Z-stacks were recorded on the upper epidermis of WT *Arabidopsis* leaves. The interval between stack slices was 1.06 μm. Maximum intensity projections were calculated from the slices spanning ∼30 μm, starting from the upper epidermis (to visualize pavement cells), or from slices spanning ∼10 μm, starting from the top of the palisade cells. Prior to quantification of the images of the epidermis, the sliding paraboloid method was used to subtract the background from the projection images, using ImageJ. Contrast was enhanced by histogram equalization. Actin fibers were marked in the images using the ridge detection method (Steger, 1998), implemented in an ImageJ plugin (Wagner et al., 2017). The areas occupied by guard cells and the contours of cells visible in the projection images were marked manually and excluded from the analysis.

### Determination of Phototropin Expression

Phototropin expression at mRNA and protein levels was examined in leaves of *Arabidopsis* WT and *uvr8* mutant plants in at least five biological replicates. Each sample contained leaves from two plants frozen in liquid nitrogen immediately after treatment. RNA isolation and real-time polymerase chain reaction (PCR) were performed as described in Łabuz et al. (2012), with the exception of the oligodT primers used for RNA reverse transcription. Primer sequences can be found in Łabuz et al. (2012) (for *PHOT1* and *PHOT2*) and in Czechowski et al. (2005) (for reference genes *UBC*, *PDF2*, and *SAND*). The relative expression of each gene in a sample was determined in three technical replicates. The mean value of *Ct* for samples from all experimental groups quantified simultaneously was subtracted from individual *Ct* values. Expression levels were then normalized using factors calculated with geNorm v3.4 (Vandesompele et al., 2002). Protein extraction was performed according to (Sakamoto and Briggs, 2002). Samples were homogenized and weighed to adjust to equal mass; 7.5% polyacrylamide gels were used for sodium dodecyl sulfate–polyacrylamide gel electrophoresis (SDS-PAGE) followed by a semi-dry transfer (Biorad). Western blot was performed as in Sztatelman et al. (2016). Anti-PHOT1 (AS10 720) and anti-PHOT2 (AS10 721) antibodies were prepared by Agrisera (see Łabuz et al., 2015). Anti-PHOT2 antibodies were used at a dilution of 1:5,000, and anti-PHOT1 antibodies at a dilution of 1:300 (a purified fraction). Secondary antibodies (goat anti-rabbit horseradish peroxidase (HRP)-conjugated IgG, Agrisera) were applied at a dilution of 1:25,000. Signal detection was performed with a Clarity Western ECL Blotting Substrate (Bio-Rad), using the BioSpectrum Imaging System (UVP Ultra-Violet Products Ltd). Intensities of the chemiluminescent signal were normalized to actin levels in each sample. Membranes were stripped with Restore Plus Western Blot Stripping Buffer (Thermo Scientific) and probed with an anti-actin antibody (AS132640, Agrisera) at a dilution of 1:2,500 at room temperature for 1 h, followed by secondary antibody incubation and enhanced chemiluminescence (ECL) detection. Densitometric quantification was performed with ImageJ.

### Statistical Analysis

Statistical calculations were performed using the R software. The mRNA and protein levels were log-transformed before statistical analysis; other measurements were not transformed. Significance of the effects of the plant line and irradiation was assessed with two-way ANOVAs. As the interaction terms were significant, the pairwise comparisons between means of groups were performed, using the *glht* command of the multcomp package. To account for unequal variances between groups, the sandwich package was used. For data shown in **Figures 1**, **2**, and **7**, as well as in **Supplementary Figures 7 and 8**, the significance of the differences of means between the control group (mock-irradiated or dark samples) and the irradiated samples was assessed for each plant line. For data in **Supplementary Figure 1D**, the differences between WT and *phot1phot2*, as well as between *glabra1* and *phot1phot2glabra1*, were tested. For data in **Supplementary Figures 2B, C**, the differences between plant lines (WT vs *jac1*) were tested for each irradiation condition. The *p* values reported with the asterisks are adjusted for multiple comparison using the Holm method. All comparisons shown in a single plot were treated as a family of comparisons for the purpose of the adjustment of *p* values.

A different procedure was used for the statistical analysis of the results of quantification of the actin cytoskeleton (shown in **Supplementary Figure 5**). In each repetition of this experiment, three similar leaves were chosen for dark control and for UV irradiation. Thus, pairs of observations can be distinguished. The means obtained for the control leaves were compared with those calculated for the UV-irradiated samples, using the paired sample *t* test in the R software. The Bonferroni correction was used to adjust the *p* values.

In all box plots, the central rectangle marks the range between the first and third quartiles, the central band is the median, and the ends of whiskers represent the lowest and greatest values. Means are marked with white circles.

## Results

To investigate the impact of UV-B on chloroplast movements in the model plant *Arabidopsis thaliana*, detached leaves of dark-adapted plants were irradiated for 1 h with UV-B of 3.3 µmol·m^−2^·s^−1^ (1.3 W·m^−2^) or strong UV-B of 20 µmol·m^−2^·s^−1^ (7.9 W·m^−2^). Control leaves were subjected to mock irradiation (darkness). Leaf transmittance was measured before and after mock/UV-B treatment with the photometric method (**Figure 1A**). The difference of transmittance ΔT was used to assess chloroplast relocations quantitatively. To identify the photoreceptor responsible for the observed effects, a set of mutants was used: *uvr8*, *phot1*, *phot2*, and *phot1phot2*. A substantial (∼2%) decrease of transmittance, indicative of chloroplast accumulation, was observed in the WT and *uvr8* leaves irradiated for 1 h with UV-B of 3.3 µmol·m^−2^·s^−1^. An even greater (3.4%) decrease in transmittance was recorded for the *phot2* mutant. No substantial changes of leaf transmittance were observed in the *phot1* and *phot1phot2* mutants. This suggests that UV-B irradiation did not induce chloroplast accumulation in those lines. To check the dose dependency of UV-B responses, *Arabidopsis* leaves were treated with higher UV-B intensity of 20 µmol·m^−2^·s^−1^ for 1 h. In WT, *uvr8*, *phot1*, and *phot1phot2* plants, the effect of UV-B irradiation on leaf transmittance was not statistically different from the effect of mock irradiation. Only in the *phot2* mutant was a larger (2%) decrease of transmittance observed. To compare the magnitude of chloroplast responses with different wavelengths, equimolar blue light (455 nm) was used (**Figure 1B**). Leaf transmittance was measured in dark-adapted WT plants and photoreceptor mutants treated with blue light of 3.3, 20, and 120 µmol·m^−2^·s^−1^, which elicits full chloroplast avoidance. In WT and *uvr8* mutant plants, treatment with blue light of 3.3 µmol·m^−2^·s^−1^ caused a 2% decrease in transmittance, which is consistent with chloroplast accumulation. The *phot1* mutant showed only a 1% decrease in transmittance, whereas the change in the *phot2* mutant was the largest, over 3%. Blue light of 20 µmol·m^−2^·s^−1^ did not induce substantial changes in transmittance of WT and *uvr8*, *phot1*, and *phot1phot2* mutant leaves. Only in in the *phot2* mutant was a large (3%) decrease of transmittance observed. Blue light of 120 µmol·m^−2^·s^−1^, which saturates chloroplast avoidance, triggered an increase in leaf transmittance in WT plants, as well as in *uvr8* and *phot1* mutants (∼6%, 5%, and 3%, respectively). In the *phot2* mutant, this blue light intensity induced a 1.5% decrease of transmittance. In the *phot1phot2* mutant, there were no statistically significant differences between the effects of blue light treatments and of mock irradiation.

UV-B intensity of 20 µmol·m^−2^·s^−1^ is much higher than the intensities occurring in field conditions, yet it is insufficient to induce chloroplast avoidance in WT *Arabidopsis* plants. This observation could be explained in two ways: strong UV-B is not capable of producing the signal to chloroplast avoidance, or it generates both signals to accumulation and avoidance, but the one leading to accumulation is prevailing. To distinguish between those scenarios, changes of leaf transmittance were investigated in the *jac1* mutant. JAC1 (J-DOMAIN PROTEIN REQUIRED FOR CHLOROPLAST ACCUMULATION RESPONSE 1) is required for signaling to chloroplast accumulation; thus, *jac1* shows chloroplast avoidance even at the blue light intensity of 1.6 µmol·m^−2^·s^−1^, which elicits chloroplast accumulation in WT plants (Suetsugu et al., 2005a) (**Supplementary Figures 2B–E**). In the *jac1* mutant, UV-B of 3.3 µmol·m^−2^·s^−1^ did not induce changes in leaf transmittance; however, UV-B treatment of 20 µmol·m^−2^·s^−1^ caused a 2% increase (**Figure 2A**). This result suggests that UV-B can trigger chloroplast avoidance, which can be observed in mutant lines defective in signaling to chloroplast accumulation. In WT plants, signaling to avoidance is masked by the prevailing accumulation response. Irradiation with blue light elicited an increase in the transmittance of *jac1* leaves. The magnitude of this increase was proportional to the light intensity (**Figure 2B**).

**Figure 3:**
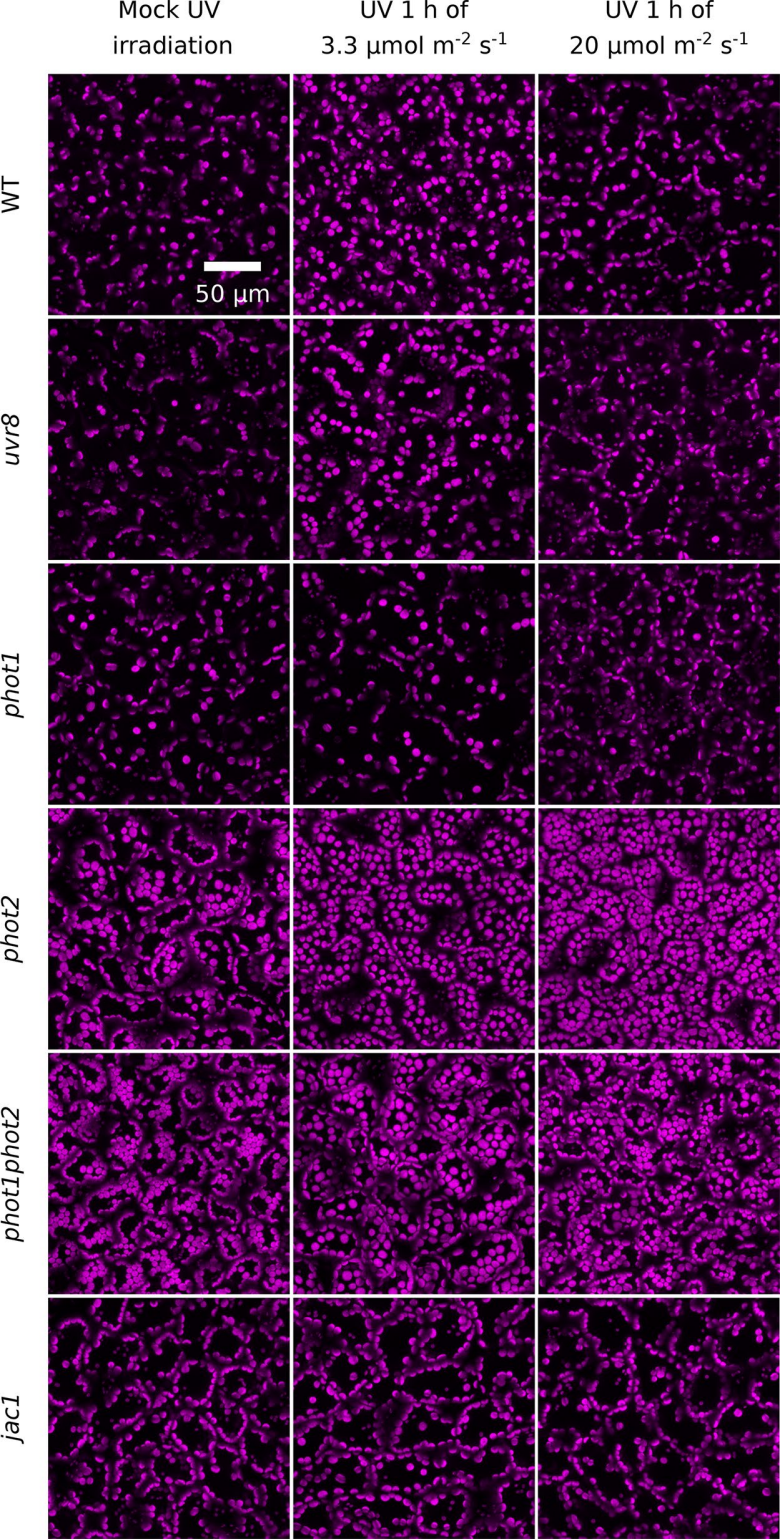
UV-B-induced chloroplast movements in palisade cells of *Arabidopsis* leaves of WT, *uvr8*, *phot1*, *phot2*, *phot1phot2*, and *jac1* mutant plants. For 1 h, leaves were kept in darkness (mock irradiation) or irradiated with UV-B (280–320 nm) of 3.3 or 20 µmol·m^−2^·s^−1^. Chloroplast arrangements were then imaged with a confocal microscope, using chlorophyll autofluorescence (in magenta). Maximum intensity projections were calculated from Z-stacks, recorded for 40 µm, starting from the leaf upper surface.

To confirm that transmittance changes resulted from chloroplast movements, chloroplast positioning was further examined under the confocal microscope in palisade mesophyll cells using chlorophyll autofluorescence (**Figure 3**). After UV-B treatment of 3.3 µmol·m^−2^·s^−1^, chloroplasts localized at the top periclinal cell walls of WT palisade cells. This relocation was not observed in leaves irradiated with UV-B of 20 µmol·m^−2^·s^−1^. The *uvr8* mutant showed similar chloroplast arrangements as WT under all experimental treatments. In the *phot1* mutant, no substantial relocations were observed in response to UV-B of 3.3 or 20 µmol·m^−2^·s^−1^. The *phot2* mutant exhibits altered dark positioning of chloroplasts (Suetsugu et al., 2005a), which form characteristic rows under the top periclinal cell walls of palisade cells (**Figure 3**). UV-B of both intensities triggered dispersion of chloroplast clusters beneath the periclinal walls. The distance between chloroplasts increased, so that they became evenly distributed. In *phot1phot2* plants, chloroplasts formed clusters beneath the top periclinal walls, and no substantial differences were observed between dark and UV-B treated leaves. The dark positioning of chloroplasts in palisade cells of the *jac1* mutant differed from the positioning in WT plants. In *jac1*, chloroplasts gathered almost exclusively at the anticlinal cell walls. UV-B treatment did not trigger chloroplast rearrangements in the upper parts of palisade cells in the *jac1* mutant (**Figure 3**). To relate microscopic observations with the results obtained by the photometric method, the area occupied by chloroplasts in the projection images was quantified (**Supplementary Figure 4**). An increase in the area occupied by chloroplasts, corresponding to chloroplast accumulation recorded with the photometric method, was observed in UV-B-irradiated WT, *uvr8*, and *phot2* leaves. The dark positioning of chloroplasts was altered in *jac1*, *phot2*, and *phot1phot2* mutants.

**Figure 4:**
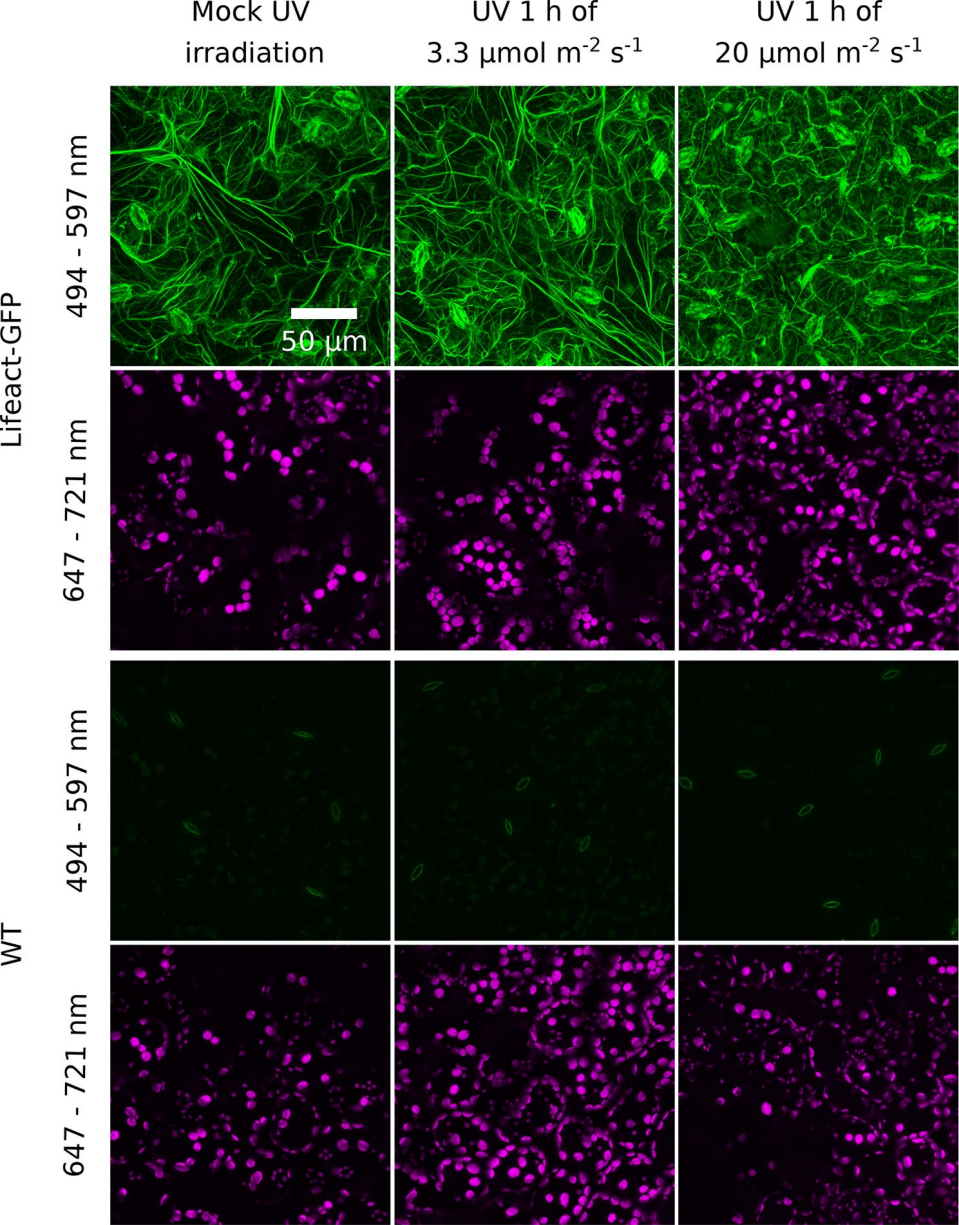
Effect of UV-B irradiation on the actin cytoskeleton visualized with Lifeact-green fluorescent protein (GFP) in the epidermal cells of *Arabidopsis* leaves. Wild-type (WT) plants serve as a control of nonspecific green fluorescence. Leaves were mock irradiated or irradiated with UV-B (280–320 nm) of 3.3 or 20 µmol·m^−2^·s^−1^ for 1 h. Cells were then imaged with a confocal microscope. The emission was recorded in the 494- to 597-nm range for GFP visualization and in the 647- to 721-nm range to visualize chloroplasts. Maximum intensity projections were calculated from Z-stacks, recorded for 30 µm, starting from the leaf upper surface.

To assess whether irradiation with UV-B has prolonged effects on chloroplast movements, we examined its impact on the actin cytoskeleton and investigated blue light-induced chloroplast movements in UV-B-pretreated leaves. *Arabidopsis* plants expressing Lifeact, an actin binding peptide fused with GFP, were exposed to different UV-B intensities and observed under the confocal microscope. Projected images were recorded for the upper epidermis (**Figure 4**) and the top parts of the palisade cells (**Supplementary Figure 6**). The cp-actin, which is specific for chloroplast movements, was too faint for analysis of the effects of UV-B. No substantial differences in the architecture of the cortical actin cytoskeleton in the pavement or palisade cells were observed between leaves kept in darkness and those irradiated with UV-B of 3.3 µmol·m^−2^·s^−1^ (**Figure 4**, **Supplementary Figure 6**). The effect of UV-B on the cortical actin cytoskeleton in the pavement cells of the upper epidermis was quantified. The average length of actin filaments and their length per area of the projection image did not differ significantly between these conditions (**Supplementary Figures 5A, B**). After treatment with strong UV-B of 20 µmol·m^−2^·s^−1^, actin filaments were still visible, but long, thick fibers were fewer, in both the pavement and palisade cells (**Figure 4**, **Supplementary Figure 6**). The level of fluorescence in the cytoplasm, the nucleus, and the vacuole increased (**Figure 4**). The results of quantification for the pavement cells indicated that the average length of actin filaments and their length per area of the projection image were smaller than in non-irradiated samples (**Supplementary Figures 5A, B**), which suggests that strong UV-B irradiation disrupted actin filaments. No cytoplasmic, vacuolar, or nuclear green fluorescence was observed in WT leaves, regardless of the conditions (**Figure 4**).

Chloroplast responses to blue light after pretreatment with UV-B of 3.3 or 20 µmol·m^−2^·s^−1^ for 1 h were examined in WT and *phot2* mutant plants using the photometric method. Blue light intensities of 1.6 µmol·m^−2^·s^−1^, eliciting chloroplast accumulation, and of 120 µmol·m^−2^·s^−1^, triggering chloroplast avoidance in WT plants, were used.

**Figure 5:**
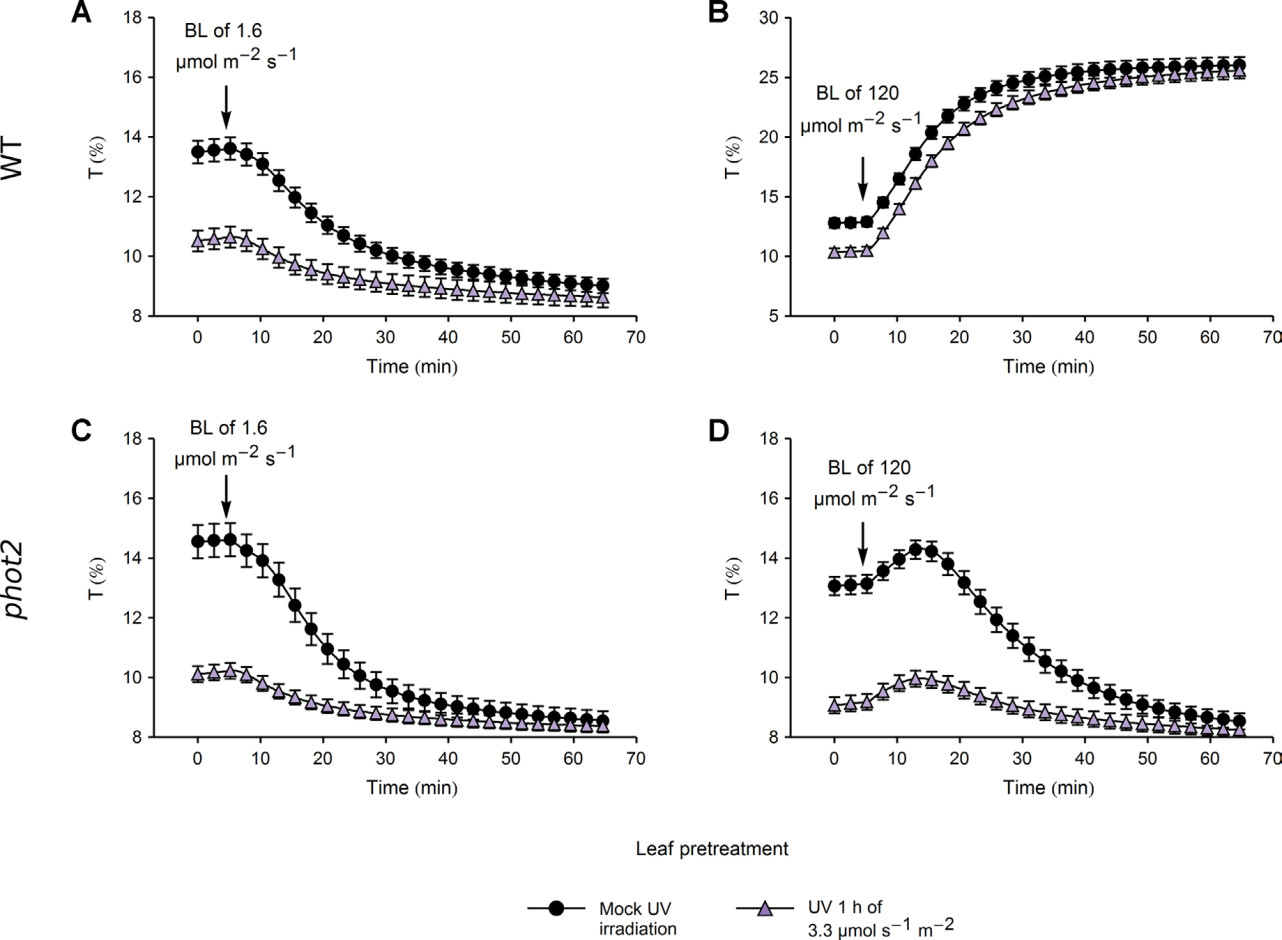
Chloroplast movements induced by blue light (BL) in control and UV-B pretreated leaves of wild-type (WT) **(A**, **B)** and *phot2* plants **(C**, **D)**. Changes in transmittance *T* induced by blue light of 1.6 **(A**, **C)** or 120 µmol·m^−2^·s^−1^ **(B**, **D)** were recorded for leaves previously irradiated with UV-B of 3.3 µmol·m^−2^·s^−1^ (triangles) or kept in darkness (disks). Each curve is an average of 20 recordings. Error bars show standard error (SE).

In WT plants, the amplitude of blue light-induced chloroplast accumulation was more than twice smaller in leaves pretreated with UV-B of 3.3 µmol·m^−2^·s^−1^ than in those kept in darkness (**Figure 5A**). However, the final transmittance was not affected by the pretreatment (**Supplementary Figure 7A**). This suggests that the small amplitude of blue light-induced accumulation in leaves irradiated with UV-B was merely due to the fact that in those leaves chloroplasts had already partially accumulated in response to UV-B. Similarly, UV-B pretreated leaves and those kept in darkness reached the same transmittance during the avoidance response induced by strong blue light of 120 µmol·m^−2^·s^−1^ (**Figure 5B**, **Supplementary Figure 7B**). In the *phot2* mutant, which shows sustained chloroplast accumulation regardless of the blue light intensity, pretreatment with UV-B of 3.3 µmol·m^−2^·s^−1^ did not affect the final transmittance after subsequent illumination with any blue light intensity (**Figures 5C, D**). Both in WT and the *phot2* mutant, the velocities of blue light-induced chloroplast accumulation were lower in leaves pretreated with UV-B of 3.3 µmol·m^−2^·s^−1^ than in the mock-irradiated ones (**Supplementary Figures 7C, D**). The differences in velocities may stem from the partial accumulation in UV-B irradiated leaves. The velocity of chloroplast avoidance in WT was not affected by the UV-B pretreatment (**Supplementary Figure 7D**).

**Figure 6:**
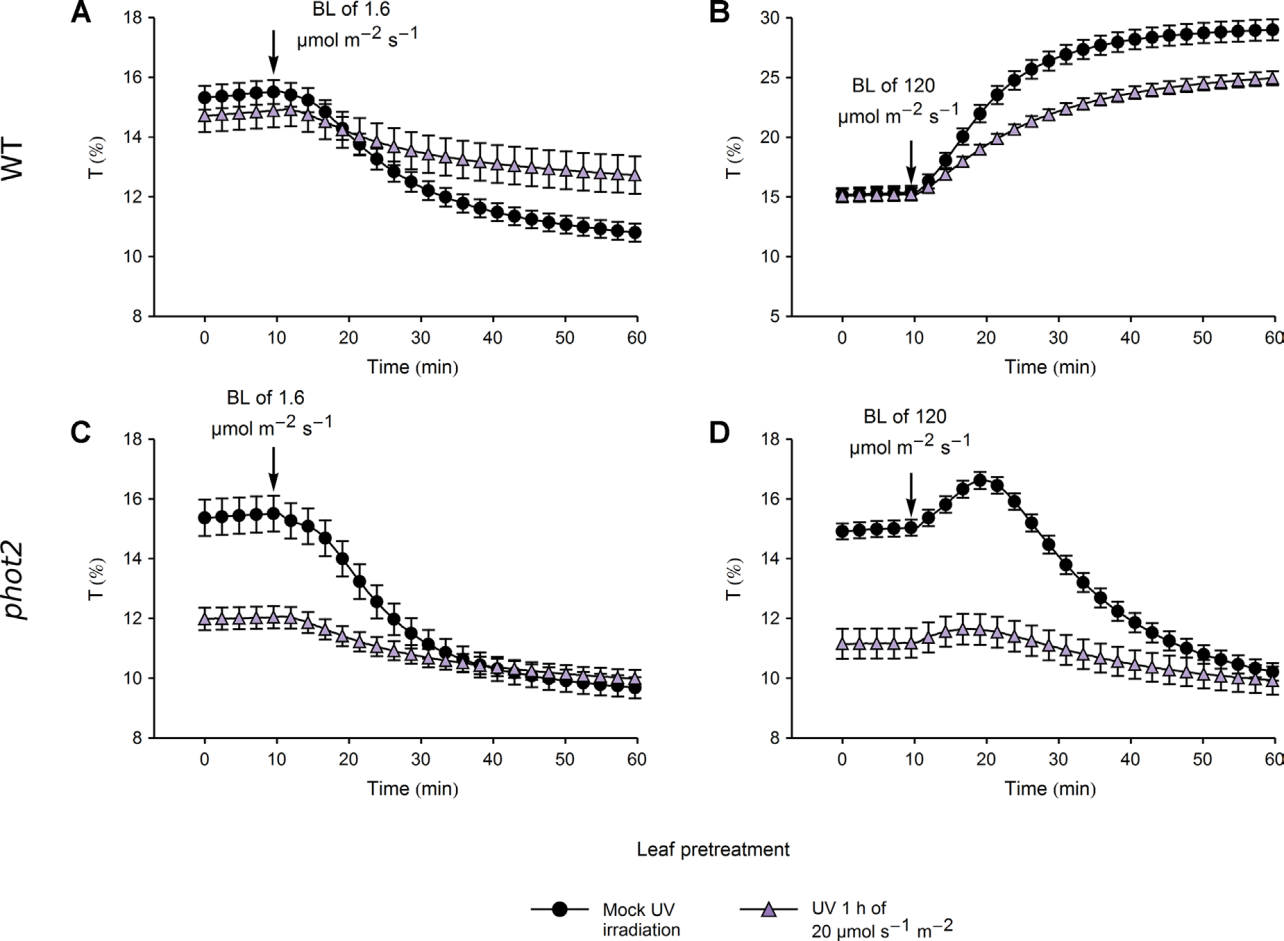
Chloroplast movements induced by blue light in control and UV-B pretreated leaves of wild-type (WT) **(A**, **B)** and *phot2* plants **(C**, **D)**. Changes in transmittance *T* induced by blue light of 1.6 **(A**, **C)** or 120 µmol·m^−2^·s^−1^ **(B**, **D)** were recorded for leaves previously irradiated with UV-B of 20 µmol·m^−2^·s^−1^ (triangles) for 1 h or mock-irradiated (disks). Each curve is an average of 14 recordings. Error bars show standard error (SE).

In WT plants, pretreatment with UV-B of 20 µmol·m^−2^·s^−1^ caused a substantial reduction in the magnitude of subsequent blue light-induced chloroplast accumulation and avoidance, even though the transmittance of UV-B and mock-irradiated leaves did not differ (**Figures 6A, B**). In line with this, the total transmittance change, calculated as the sum of transmittance changes induced by pretreatment and blue light illumination, differed significantly between the UV-B and mock-irradiated leaves (**Supplementary Figures 8A, B**). This indicates a nonspecific attenuation of chloroplast movements by strong UV-B. In the *phot2* mutant, the transmittance reached during blue light-induced chloroplast accumulation did not differ between leaves pretreated with strong UV-B and the mock-irradiated ones (**Supplementary Figures 8C, D**). However, the velocities of chloroplast responses to blue light were substantially diminished in WT and *phot2* leaves pretreated with strong UV-B (**Supplementary Figures 8C, D**).

**Figure 7:**
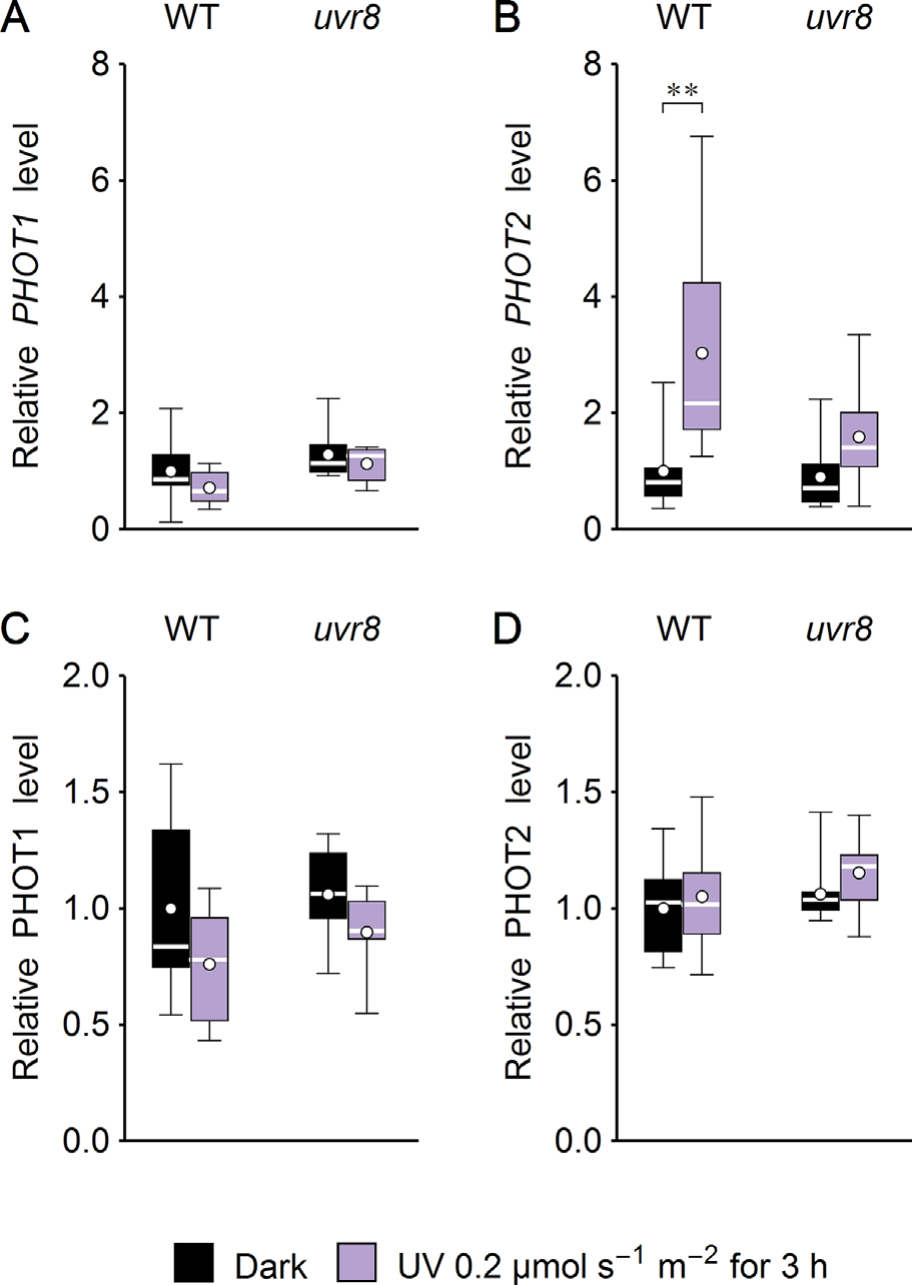
Effect of UV on the expression of phototropins in *Arabidopsis* leaves of wild type (WT) and the *uvr8* mutant. Whole plants were irradiated for 3 h with UV of 0.2 µmol·m^−2^·s^−1^. The expression of phototropin1 **(A**, **C)** and phototropin2 **(B**, **D)** was examined at the mRNA level, using quantitative real-time polymerase chain reaction (PCR) **(A**, **B)** and immunoblotting followed by densitometry **(C**, **D)**. Each box represents 7 and 8 **(A**, **B)**, 7 **(C)**, or 10 **(D)** biological replicates. Asterisks indicate statistically significant differences between illuminated and dark-adapted samples (adjusted *p* values: **0.001 < *p* < 0.01).

Several studies suggest that chloroplast movements depend on the expression level of the photoreceptor. Phot1-controlled chloroplast accumulation depends on its protein level (Doi et al., 2004). The velocity of chloroplast avoidance depends on phot2 protein expression (Kagawa and Wada, 2004; Kimura and Kagawa, 2009). To check whether UV-B irradiation may also affect chloroplast movements indirectly by controlling the amount of photoreceptors, phototropin1 and phototropin2 expression was investigated at the mRNA and protein levels in *Arabidopsis* leaves (**Figure 7**). WT and *uvr8* plants were either treated with UV of 0.2 µmol·m^−2^·s^−1^ to induce the UVR8-regulated gene expression pathway (Brown and Jenkins, 2007) or kept in darkness for 3 h. UV slightly decreased phototropin1 expression at the mRNA and protein levels (**Figures 7A, C**) in both plant lines. *PHOT2* mRNA level was up-regulated by UV in WT plants. This effect was less prominent in the *uvr8* mutant (**Figure 7B**). At the protein level, the impact of UV treatment was observed neither in WT nor in *uvr8* mutant plants (**Figure 7D**).

## Discussion

UV-B induces chloroplast directional movements in the model plant *Arabidopsis thaliana*. Irradiation with UV-B of 3.3 µmol·m^−2^·s^−1^ triggers substantial chloroplast accumulation in WT, *uvr8*, and *phot2* plants, but not in *phot1* and *phot1phot2* mutants (**Figure 1A**). Chloroplast responses to equimolar blue light are similar (**Figure 1B**). Chloroplast accumulation induced by UV-B relies on the presence of a protein necessary for blue light-induced accumulation, JAC1 (**Figure 2A**). JAC1 is responsible for inhibiting chloroplast avoidance triggered by phot2, as in strong blue light, the phenotype of the *phot2jac1* double mutant resembles that of the *phot2* single mutant (Kodama et al., 2010). Dependence of both UV-B- and blue light-induced chloroplast accumulation on JAC1 suggests that the signaling pathways used for these responses are the same or at least share some of their components. Chloroplast accumulation after UV-B of 3.3 µmol·m^−2^·s^−1^ is more prominent in the *phot2* mutant than in WT (**Figure 1A**). This parallels the observations of increased blue light sensitivity of this mutant in the chloroplast accumulation response (Luesse et al., 2010). The enhancement of phot1-mediated chloroplast accumulation after blue light pulses, observed in the absence of phot2, was described in Sztatelman et al. (2016). This effect may result from physical interactions of phototropins. It appears that the presence of phot2 attenuates chloroplast accumulation triggered by short pulses of strong blue light in WT plants. In the *phot2* mutant, only phot1 is present, so the attenuation is not observed. Phototropin interactions, predominantly through their N-terminal parts, may be responsible for this effect, as phototropin levels remain unaffected (Sztatelman et al., 2016). It appears that the postulated ability of phototropins to form homodimers and heterodimers influences chloroplast responses to UV-B. Similarities between UV-B- and blue light-induced movements are also apparent when high intensities are used. UV-B of 20 µmol·m^−2^·s^−1^ induces chloroplast accumulation only in the *phot2* mutant (**Figure 1A**). Likewise, blue light of the same intensity induces accumulation only in this mutant (**Figure 1B**).

Although the results of quantification of microscopic images (**Supplementary Figure 4**) roughly agree with the results obtained with the photometric method, direct comparison is not straightforward. Microscopic observations are performed for palisade cells. The photometric method detects chloroplast relocations throughout the leaf tissue, including cell layers that are located deeper in the mesophyll and thus receive less light. The gradient of UV-B in the tissue is probably much higher than that of visible light, due to its increased absorption. In *Funaria*, the transmittance of the whole cell (excluding chloroplasts) is high (∼90%) in the visible range and UV longer than 330 nm. At shorter wavelengths, the transmittance is reduced, decreasing to 62% at 300 nm (Zurzycki, 1967).

The increase in leaf transmittance upon strong UV-B irradiation observed in the *jac1* mutant indicates that UV-B can elicit also chloroplast avoidance (**Figure 2A**). Only phot2 is capable of mediating sustained avoidance. It seems to be activated by UV-B, though its sensitivity in this range is lower than to blue light (**Figure 1**). Thus, both phototropins may trigger chloroplast relocations upon UV-B irradiation. In field conditions, UV-B intensity does not exceed 3 W·m^−2^ (Aphalo et al., 2012). If no other radiation is present, such intensities of UV-B induce only chloroplast accumulation in WT *Arabidopsis* plants. It may seem counterintuitive that the potentially protective chloroplast avoidance cannot be induced in WT plants even by strong UV-B, which induces oxidative stress and directly causes DNA lesions. However, in the field, UV-B is always accompanied by UV-A and blue light, and thus, those different wavelengths probably act additively to elicit chloroplast relocations. The intensity of UV-B is usually much smaller than the intensity of accompanying UV-A and blue light. The avoidance response is sensitive to UV-A and blue light, which may have reduced the selective pressure for UV-B-induced avoidance. Low-intensity UV-B can act as an environmental signal. Chloroplast movements are induced mainly in plants that grow in changing light conditions, not in full sunlight (Augustynowicz and Gabryś, 1999; Higa and Wada, 2016). The UV-B to UV-A ratio changes depending on the light conditions and is higher in the tree shade as compared with full sunlight (Parisi and Kimlin, 1999). This suggests that under the canopy, the activation of phototropins by the UV-B part of the spectrum may contribute to the induction of chloroplast accumulation and to optimization of photosynthesis.

The differences in chloroplast responses to blue light observed between leaves pretreated with UV-B of 3.3 µmol·m^−2^·s^−1^ and mock-irradiated ones (**Figure 5**) can be explained by the initial, UV-B induced movement of chloroplasts towards the periclinal cell walls. This is not the case when leaves are pretreated with strong UV-B of 20 µmol·m^−2^·s^−1^ (**Figure 6**). In WT plants, initial transmittance changes do not differ between UV-B and mock-irradiated samples, but chloroplast responses to blue light of 1.6 (**Figure 6A**) and 120 µmol·m^−2^·s^−1^ (**Figure 6B**) are diminished, in terms of both amplitudes and rates (**Supplementary Figure 8**). This indicates that strong UV-B impairs both chloroplast accumulation and avoidance in an unspecific manner, probably due to cell damage. Microscopic examination of the actin cytoskeleton in epidermal cells (**Figure 4**) and the upper parts of the palisade cells (**Supplementary Figure 6**) supports this assumption. In wheat protoplasts, UV-B irradiation disturbs the actin cytoskeleton, which forms foci at different stages of the cell cycle. Those effects are accompanied by the formation of apoptotic bodies (Chen and Han, 2016). UV-B-induced programmed cell death has also been reported for BY2 cells (Lytvyn et al., 2010). Thus, chloroplast movement inhibition after strong UV-B may result from actin disruption caused not directly by irradiation but by programmed cell death induction. On the other hand, strong UV-B intensity used in this study still induces chloroplast accumulation in the *phot2* mutant (**Figure 1A**) and avoidance in *jac1* (**Figure 2A**).

Our results indicate that UVR8 is not involved in controlling chloroplast movements under UV-B (**Figures 1A** and **3**). This agrees with its predominantly nuclear activity and relatively low expression level in the leaf mesophyll (Bernula et al., 2017). UVR8 enters the nucleus after UV-B treatment (Kaiserli and Jenkins, 2007), where it controls gene expression. Its role in the cytoplasm is not well understood. UVR8 is capable of triggering phototropism in seedlings in the absence of phototropins (Vandenbussche et al., 2014). However, phototropism relies on a gradient formation of signaling components across the whole tissue (Morrow et al., 2018), which can be obtained even with a photoreceptor localized mainly in the nucleus, such as UVR8. Chloroplast movements are very local. They can be induced in a small volume of the cell by microbeam irradiation and require photoreceptors that are spatially oriented inside the cell through their association with the plasma membrane (Sakamoto and Briggs, 2002; Kong et al., 2013a) or the chloroplast envelope (Kong et al., 2013b). Our results suggest that low-intensity UV up-regulates *PHOT2* mRNA levels, and this process is at least partially mediated by the UVR8 photoreceptor (**Figure 7B**). However, UV-B has no impact on phototropin protein levels (**Figures 7C, D**).

We demonstrate that the UV-B part of the spectrum takes part in the regulation of chloroplast movements in *Arabidopsis* in a manner dependent on phototropins. Our results suggest that phototropin1 is required for efficient UV-B-induced chloroplast accumulation. The involvement of phototropins in UV-B sensing has been shown previously for phototropism (Vandenbussche et al., 2014; Vanhaelewyn et al., 2016). Perception of UV-B by phototropins may be important for plants growing under the canopy, where higher UV-B to UV-A ratios are observed. Thus, the phototropin pathway may contribute to the physiological effects of UV-B. However, the UV-B-induced chloroplast accumulation described in this work differs from typical responses to UV-B, mediated by UVR8, which include modulation of development and induction of mechanisms that protect the plant against damage. Our results add new insights into the diversity of plant responses to UV-B and its potential applications.

## Data Availability Statement

All datasets generated for this study are included in the manuscript/**Supplementary Files**.

## Author Contributions

PH, JŁ, and AKB designed the study. PH, JŁ, AKB, and OS performed the experiments. JŁ wrote the manuscript. PH, AKB, OS, and HG commented and improved the manuscript.

## Conflict of Interest

The authors declare that the research was conducted in the absence of any commercial or financial relationships that could be construed as a potential conflict of interest.
