# Supplementary material for: UV-B Induces Chloroplast Movements in a Phototropin-Dependent Manner
Source: Front Plant Sci. 2019 Oct 15;10:1279. doi: 10.3389/fpls.2019.01279 (PMC6804469; doi:10.3389/fpls.2019.01279)
Supplement: Supplementary file 1 [file DataSheet_1.pdf]

Supplementary Data

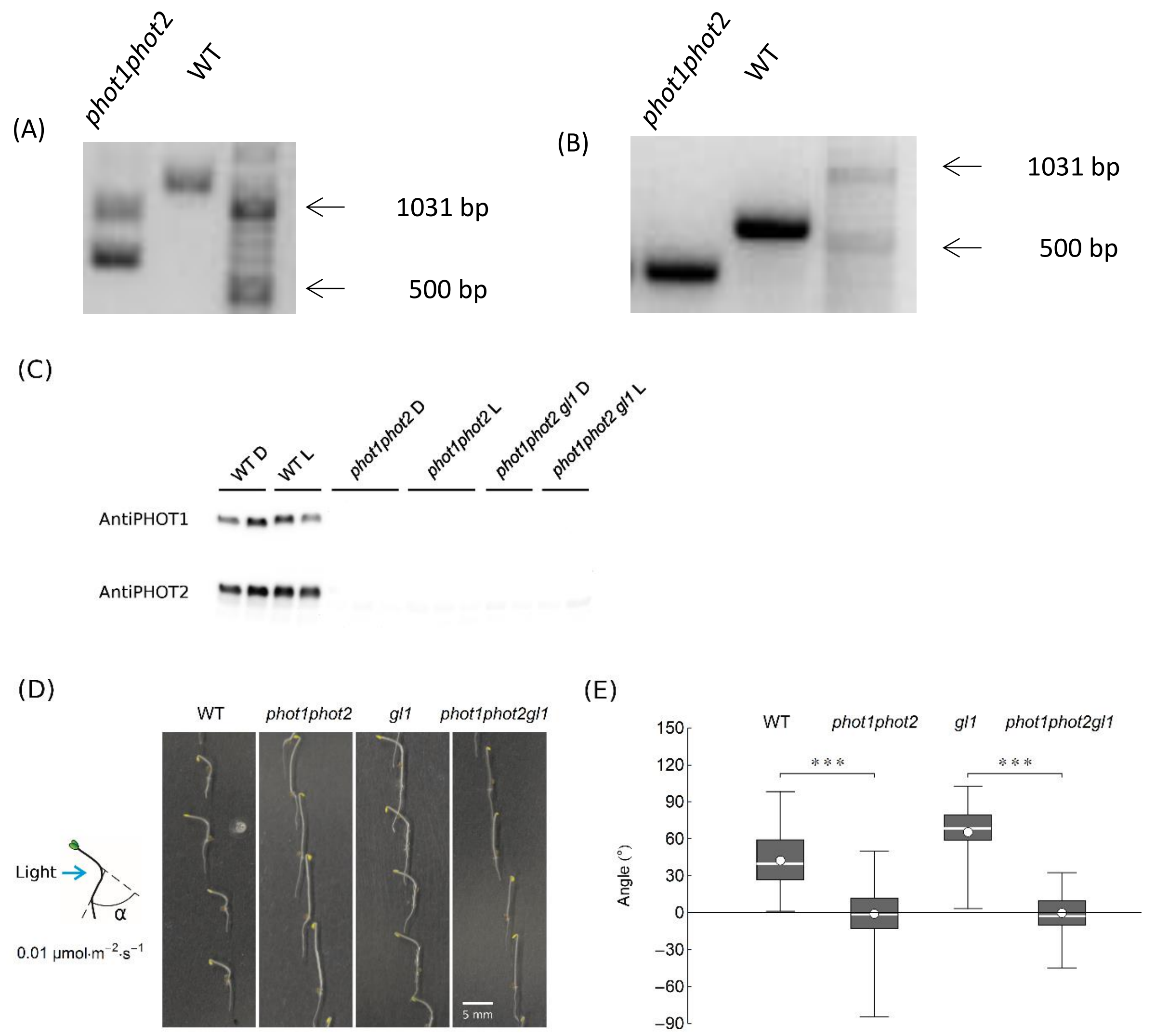

Supplementary Figure 1. Characterization of the *phot1phot2* double mutant selected from crosses. Confirmation of (A) the *phot1* mutation with: LP: 5'-TCGAACATTTCTTTGCAAATTC-3', RP: 5' - TCATCCAAAGATTCGCTCTTC - 3' and LBb1: 5'-ATTTTGCCGATTTCGGAAC - 3' primers. The predicted product size for WT: 1164 bp, the product size for the *PHOT1* (SALK_088841) mutation: 540-840 bp (calculated by T-DNA Primer Design tool: http://signal.salk.edu/tdnaprimers.2.html), (B) the *phot2* mutation with: Phot2FOR: 5-GACGCTACACAGCCTCACTGTCCC- 3', Phot2REV: 5' - CAGATACCATCATATCGAATCAAG - 3' and LBb1: 5'-ATTTTGCCGATTTCGGAAC - 3' primers. The product size for the WT: 581 bp. DNA was separated in 1% agarose in TAE buffer and stained with Midori Green. (C) PHOT1 and PHOT2 protein levels in dark-adapted rosette leaves of *Arabidopsis* WT, the *phot1phot2* mutant used in this study and a *phot1phot2* mutant in the *glabra1* (*gl1*) background, either kept in darkness (D) or irradiated with white light (L) of 120 $\mu mol \cdot m^{-2} \cdot s^{-1}$ for 3 h. Protein extracts were analyzed by Western blotting with anti-PHOT1 (AS10 720) and anti-PHOT2 (AS10 721) antibodies obtained from Agrisera. (D) Examples of 3-day-old seedlings used for calculation of phototropic bending after 12 h-long treatment with unidirectional blue light of 0.01 $\mu mol \cdot m^{-2} \cdot s^{-1}$ (spectrum in the Supplementary Fig. 3B). Before the treatment, *Arabidopsis* WT, *glabra1* and phototropin double mutant seedlings were sown on MS agar plates, kept in 4°C for 2 days, irradiated with white light for 2 h and grown vertically in darkness for 3 days. The drawing shows how the bending angle of seedlings is defined. (E) The angle

of phototropic bending measured on 3-day-old etiolated seedlings after 12 h-long treatment with blue light of 0.01 µmol·m^{-2}·s^{-1}. Asterisks indicate significant differences between the mutant and control lines (*** - adjusted $p < 0.001$). Each box represents measurements performed on 93-104 seedlings.

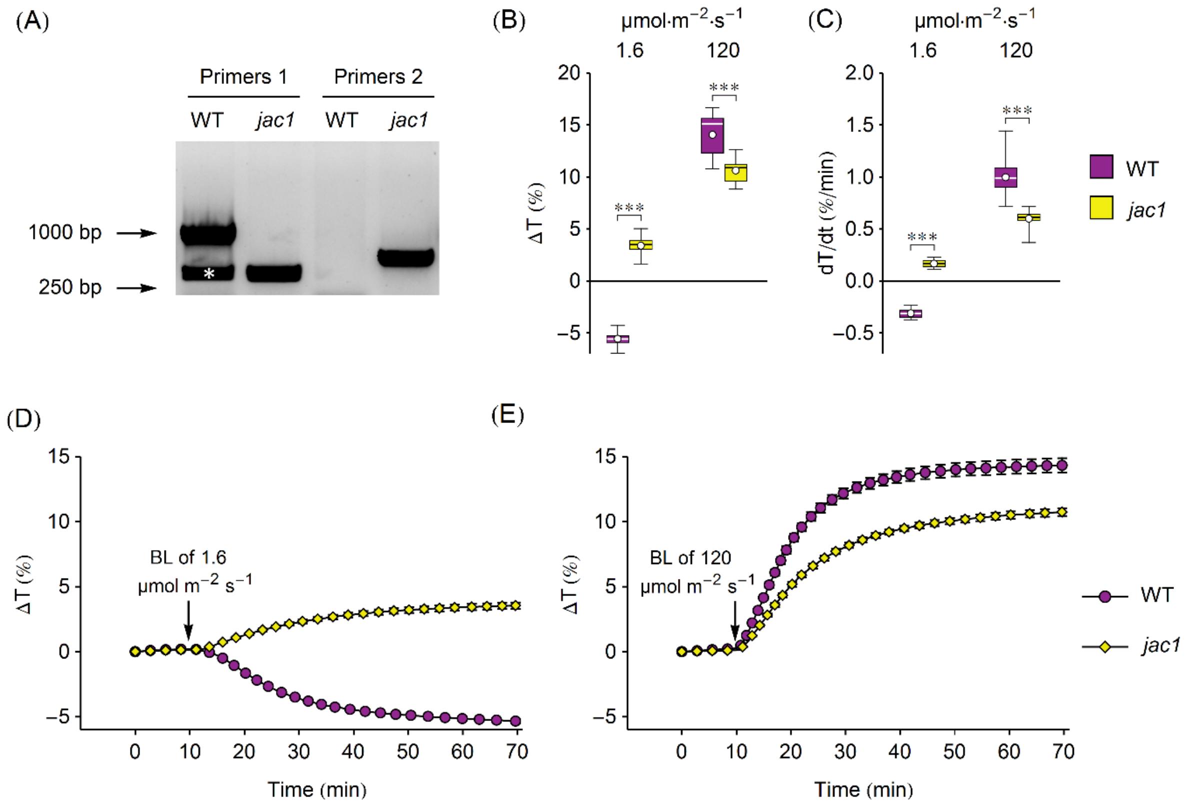

Supplementary Figure 2. Characterization of the *jac1* mutant. (A) Confirmation of *jac1-3* mutation (WiscDsLox457-460P9, insertion in the second exon of *AT1G75100*) with the primer pair 1: WiscDsLox457-460P9_LP 5' - ACATGTCTGCAGAAACCAACC - 3' and WiscDsLox457-460P9_RP 5' – GTGGACATCGATTTTGGTGAC – 3' or with the primer pair 2: LB_WiscDsLox: 5'-AACGTCCGCAATGTGTTATTAAGTTGTC - 3' and WiscDsLox457-460P9_RP 5' – GTGGACATCGATTTTGGTGAC – 3'. The predicted product size for WT: 1051 bp, the product size for the *jac1* mutation: 539-839 bp (calculated by T-DNA Primer Design tool: http://signal.salk.edu/tdnaprimers.2.html). DNA was separated in 0.8% agarose in TAE buffer and stained with ethidium bromide. The asterisk indicates a non-specific product for the genotyping primer pair. (B, C) Amplitudes ΔT (B) and maximal rates dT/dt (C) of chloroplast responses to continuous blue light of 1.6 or 120 µmol·m^{-2}·s^{-1} in leaves of dark-adapted plants. Each box represents measurements on 14 leaves. Asterisks indicate statistically significant differences between means for WT and *jac1* (*** - adjusted $p < 0.001$). (D, E) Averaged curves of changes in leaf transmittance T induced by blue light of (D) 1.6 µmol·m^{-2}·s^{-1} and (E) 120 µmol·m^{-2}·s^{-1} in WT and the *jac1-3* mutant obtained in this study. The curves used to calculate parameters presented in B and C. Arrows indicate the onset of blue light. Error bars represent SE.

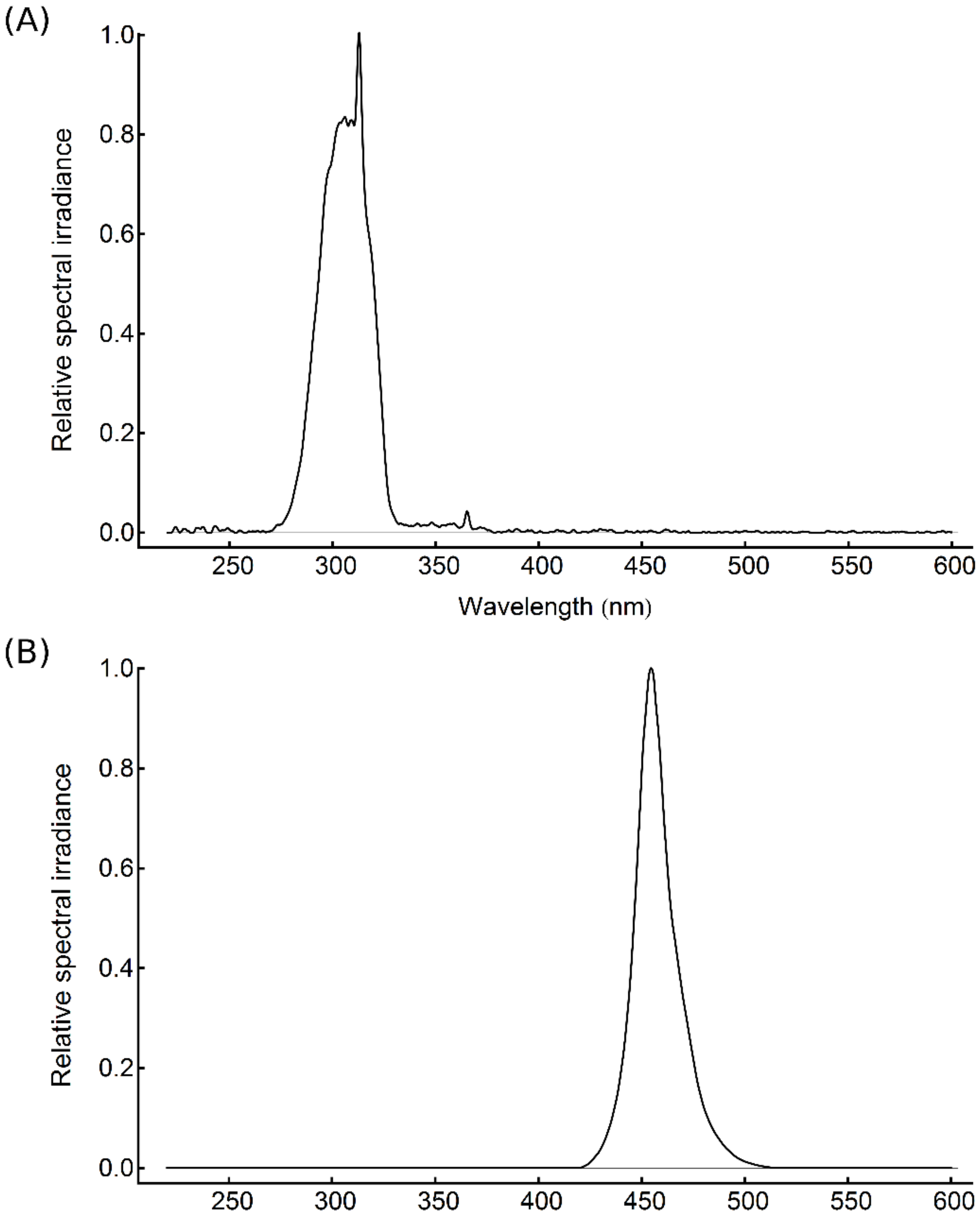

Supplementary Figure 3. Spectra of radiation used to induce chloroplast relocations. (A) Radiation emitted by USHIO UV-B G8T5E fluorescent tubes, filtered through UG-11 (Knight Optical, UK), ZUS0325 (Asahi Spectra Co, Japan) filters and two layers of cellulose acetate foil (95 µm thick, Rachow Kunststoff-Folien, Germany). (B) Light emitted by blue LEDs (LXHL-PR09, Ledium Ltd., Hungary). The maxima of the spectra were set to 1. Spectra were measured with a Black Comet C spectrometer (Stellar Net, USA).

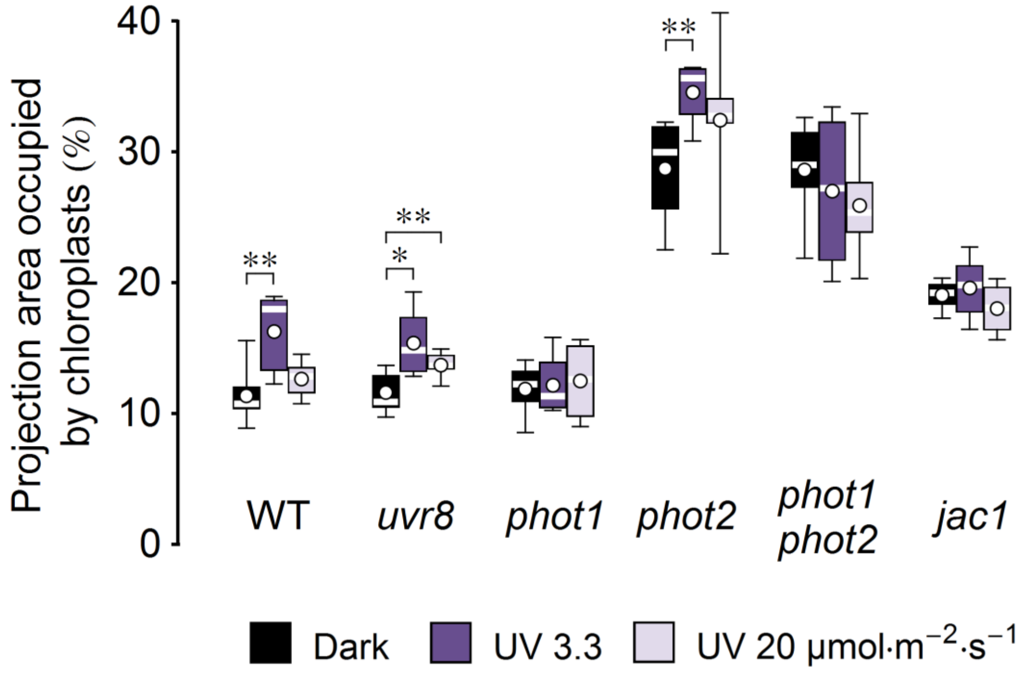

Supplementary Figure 4. Fraction of the area occupied by chloroplasts in projection images of palisade cells, recorded on *Arabidopsis* leaves of WT, *uvr8, phot1, jac1, phot2* and *phot1phot2* mutant plants. Leaves were irradiated for 1 h with UV-B (violet boxes) of 3.3 µmol·m^{-2}·s^{-1} or 20 µmol·m^{-2}·s^{-1} or kept in darkness (black boxes). Maximum intensity projections were calculated from Z-stacks, spanning 40 µm, starting from the leaf upper surface. Each box represents measurements recorded from at least 4 leaves. Three stacks were recorded on every leaf and the mean value of the area calculated from three stacks was treated as a single measurement. Asterisks indicate statistically significant differences between means for WT and mutant lines (adjusted p values: * - $0.01< p <0.05$, ** - $0.001<p < 0.01$).

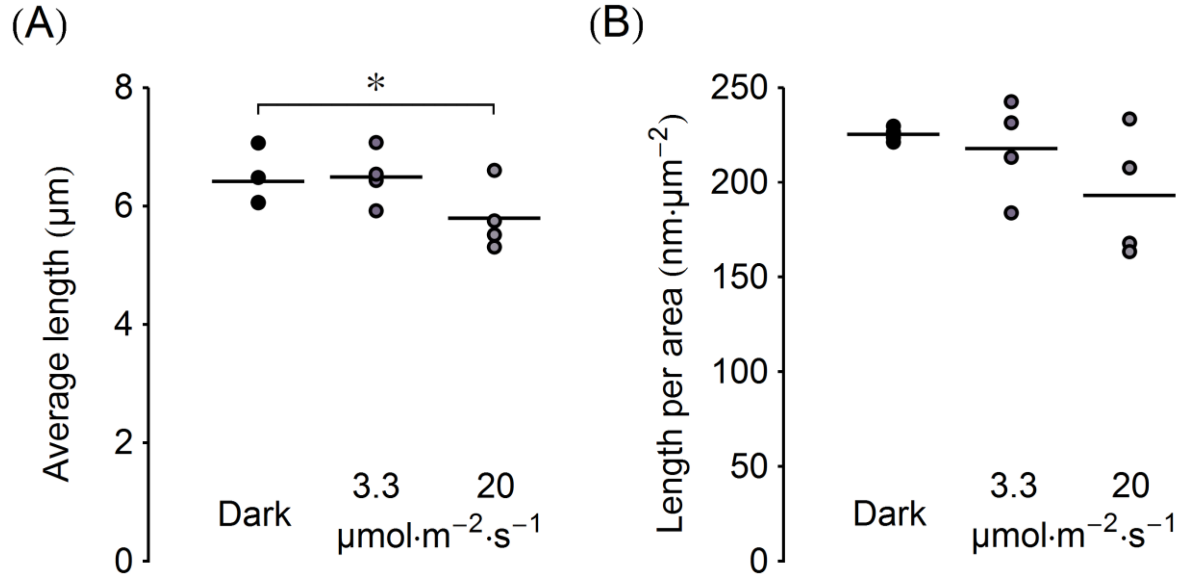

Supplementary Figure 5. Effect of UV-B irradiation on the average length (A) and length per area of the projection image (B) of actin fibers visualized with Lifeact-GFP in pavement cells of *Arabidopsis* leaves shown on Figure 4. Leaves were mock-irradiated or irradiated with UV-B (280 – 320 nm) of 3.3 µmol·m^{-2}·s^{-1} or 20 µmol·m^{-2}·s^{-1} for 1 h. Maximum intensity projections were calculated from Z-stacks, recorded for 30 µm, starting from the leaf upper surface. Each dot represents the mean value obtained by

quantification of three stacks, recorded on a single leaf. For each treatment, stacks obtained on four leaves were quantified. Horizontal bars represent means of all measurements for a treatment. Asterisks indicate statistically significant differences between means for different treatments (adjusted p values: * - 0.01< p <0.05).

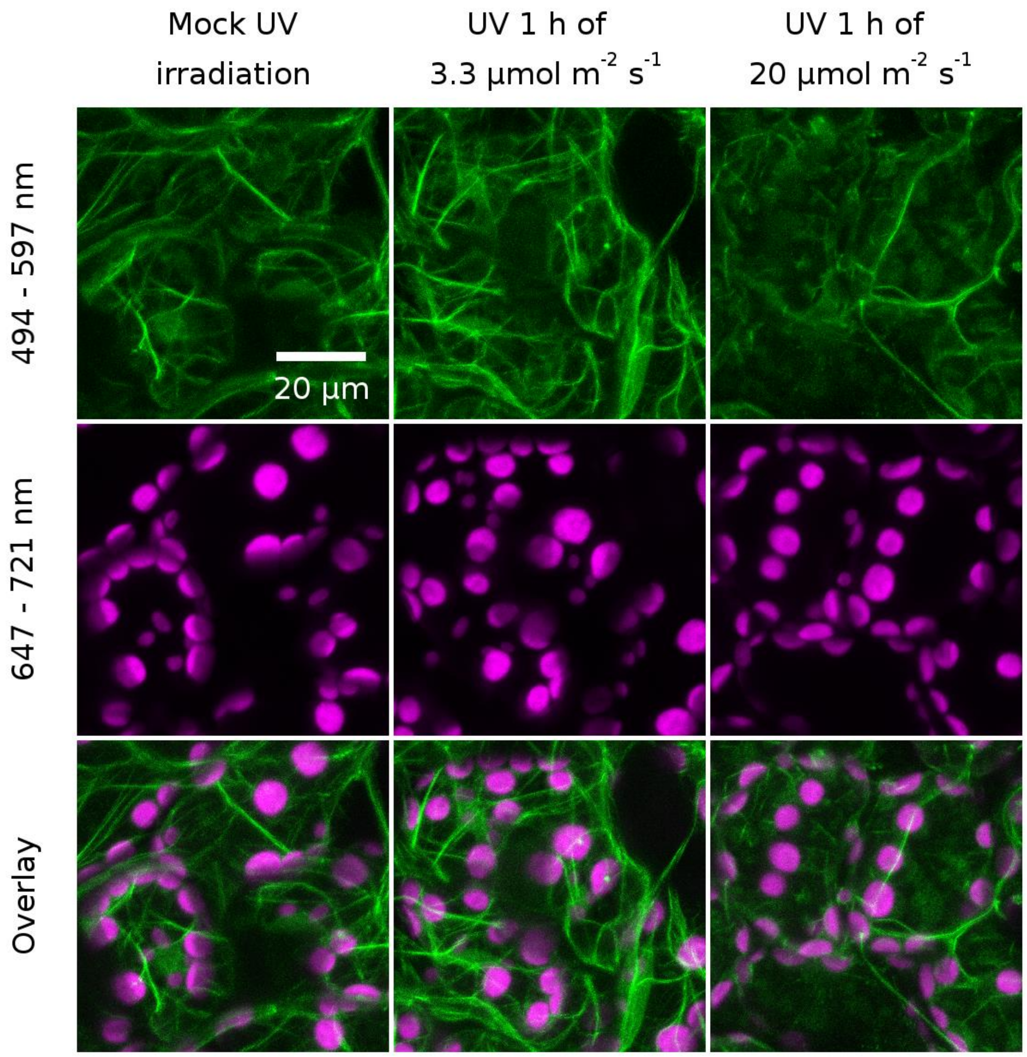

Supplementary Figure 6. Effect of UV-B irradiation on the actin cytoskeleton visualized with Lifeact-GFP in the palisade cells of *Arabidopsis* leaves. Leaves were mock-irradiated or irradiated with UV-B (280 – 320 nm) of 3.3 µmol·m^{-2}·s^{-1} or 20 µmol·m^{-2}·s^{-1} for 1 h. Palisade cells were then imaged with a confocal microscope through the intact epidermis. The emission was recorded in the 494 – 597 nm range for GFP visualization and in the 647 – 721 nm range to visualize chloroplasts. Maximum intensity projections were calculated from slices of Z-stacks, corresponding to ca. 10 µm, starting from the top of the palisade cells.

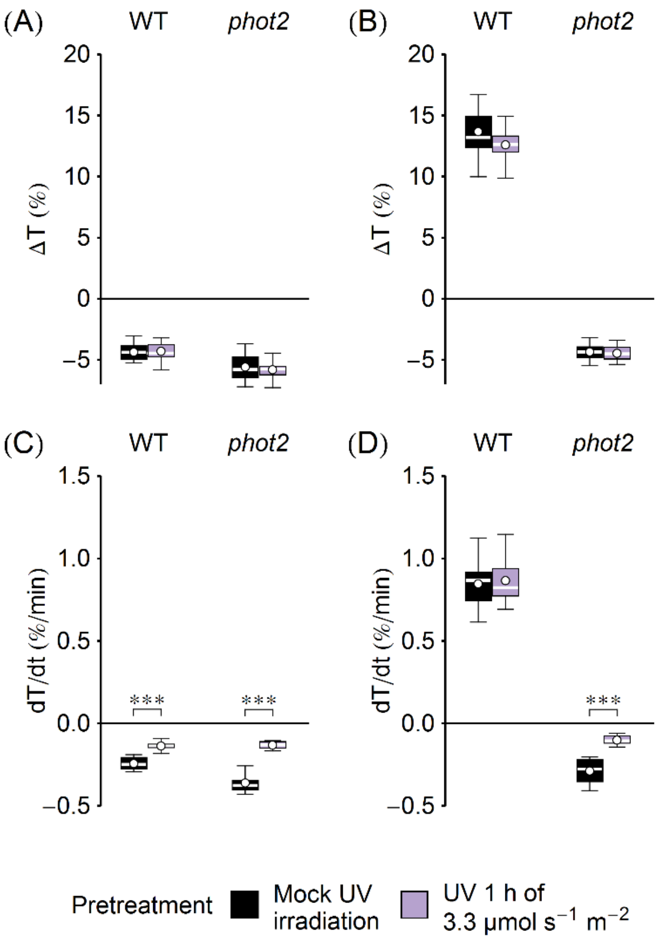

Supplementary Figure 7. (A, B) The total amplitudes ΔT of chloroplast responses induced by irradiation with UV-B of 3.3 µmol·m^{-2}·s^{-1} for 1 h and subsequent irradiation with blue light of 1.6 (A) or (B) 120 µmol·m^{-2}·s^{-1} for 1 h. (C, D) The maximal rates dT/dt of chloroplast movements induced by blue light of 1.6 µmol·m^{-2}·s^{-1} (C) or 120 µmol·m^{-2}·s^{-1} (D) in leaves of WT and *phot2* plants pre-treated with UV-B of 3.3 µmol·m^{-2}·s^{-1} for 1 h. Each box corresponds to 18 – 20 measurements. Asterisks indicate statistically significant differences between means for leaves pretreated with UVB and the mock-irradiated ones (*** - adjusted $p < 0.001$).

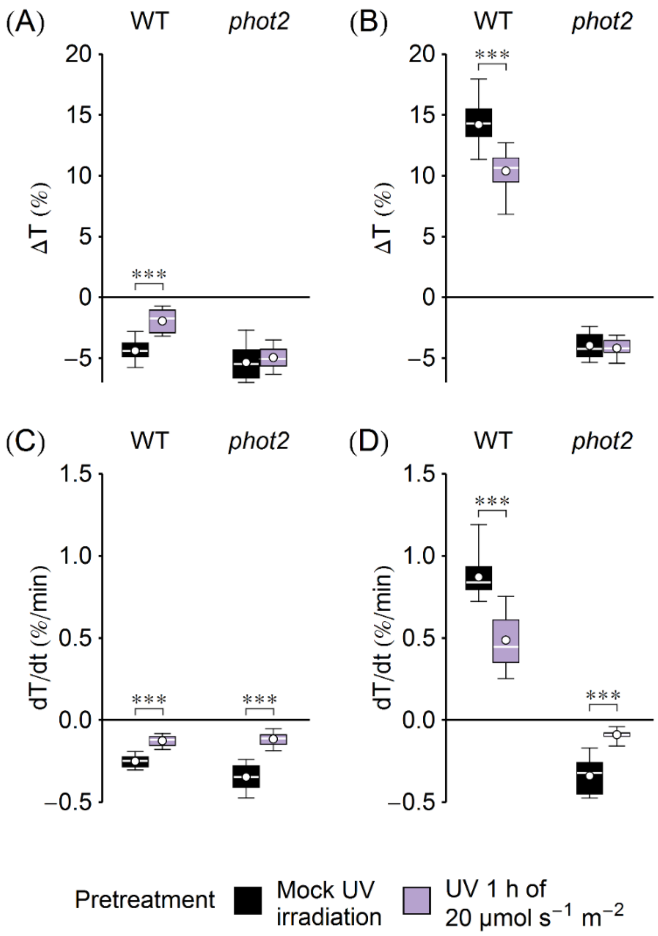

Supplementary Figure 8. The total amplitudes ΔT of chloroplast responses induced by irradiation with UV-B of 20 µmol·m^{-2}·s^{-1} for 1 h and subsequent irradiation with blue light of 1.6 (A) or (B) 120 µmol·m^{-2}·s^{-1} for 50 min. (C, D) The maximal rates dT/dt of chloroplast movements induced by blue light of 1.6 µmol·m^{-2}·s^{-1} (C) or 120 µmol·m^{-2}·s^{-1} (D) in leaves of WT and *phot2* plants pre-treated with UV-B of 20 µmol·m^{-2}·s^{-1} for 1 h. Each box corresponds to 14 measurements. Asterisks indicate statistically significant differences between means for leaves pretreated with UVB and the mock-irradiated ones (*** - adjusted $p < 0.001$).
